# Black sand nanoparticles and heat stress impacts the neurological and oxidative stress indices and splenic-renal histology of *Clarias gariepinus*

**DOI:** 10.1038/s41598-024-71707-6

**Published:** 2024-09-23

**Authors:** Alaa El-Din Hamid Sayed, Rashad E. M. Said, Mohamed Abd El-Aal, Eman Saad, Walied A. Kamel, Mohamed Hamed

**Affiliations:** 1 Department of Zoology, Faculty of Science, Assiut University, Assiut, 71516 Egypt; 2 Molecular Biology Research & Studies Institute, Assiut University, Assiut, 71516 Egypt; 3 Department of Zoology, Faculty of Science, Al-Azhar University (Assiut Branch), Assiut, 71524 Egypt; 4 Chemistry Department, Faculty of Science, Assiut University, Assiut, 71516 Egypt; 5 Department of Geology, Faculty of Science, Assiut University, Assiut, 71516 Egypt; 6 Department of Zoology, Faculty of Science, Mansoura University, Mansoura, 35516 Egypt; 7 Laboratory of Fundamental Oncology, National Cancer Center Research Institute, Tsukiji 5-1-1, Chuo-Ku, Tokyo, 104-0045 Japan; 8 Department of Comparative Biomedical Sciences, School of Veterinary Medicine, Louisiana State University, Skip Bertman Drive, Baton Rouge, LA 70803 USA

**Keywords:** BS-NPs toxicity, Thermal stress, Catfish, Environmental nanoparticles, Neurochemical alterations, Ecology, Zoology, Biogeochemistry, Environmental sciences

## Abstract

In Egypt, while many studies have focused on the radiometry and mineralogy of black sands, research on their effects on nearby aquatic organisms is rare. This study aimed to assess the combined effects of heat stress (HS) and black sand nanoparticles (BS-NPs) on renal function, antioxidant responses (TAC, SOD, CAT), neuro-stress indicators (AchE, cortisol), and to conduct histopathological investigations in the kidney and spleen tissues of African catfish *Clarias gariepinus* over a 15-day period to exposure to control, HS (32 °C), BS (6.4 g/kg diet) and HS + BS groups. The outcomes revealed that thermal stress alone showed no significant difference from the control. However, creatinine and uric acid levels were significantly higher in the BS-NPs and HS + BS-NPs groups (p < 0.001). Antioxidant markers (TAC, SOD, and CAT) were substantially reduced across all treated groups (0.05 ≥ p < 0.0001). AchE levels were significantly elevated in BS-NPs and HS + BS-NPs (p < 0.001), while cortisol levels were higher in these groups but not significantly different in HS. Degeneration and necrosis in the white and red pulps, scattered lymphocytes, and increased collagen fiber surrounding blood vessels and the lining of the ellipsoid structure were all evident in the spleen, along with the enlargement of the melanomacrophage centers with big granular, irregular, and brown pigments (hemosiderin). Our study, therefore, provides new insights into how heat stress, an abiotic environmental factor, influences the toxicity of black sand nanoparticles in catfish.

## Introduction

Impacts resulting from human activity, such as the accumulation of greenhouse gases emissions, have drastically contaminated the environment and clearly aided in global warming and heat stress^1,2^, and warmer water can negatively impact aquatic life and the aquatic ecosystem^3,4^. Fish may experience physiological problems or may die if the temperature goes above what is considered unsafe for that species^5^. Research on the consequences of heat stress on aquatic animals, including fish, has been conducted in this context. Experimentally, the research conducted by Breckels and Neff^5^ explored the impact of projected global warming patterns on the survival and sexual characteristics of the Trinidadian guppy. The study conducted by Lau et al.^6^ investigated the acute toxicity levels of nine distinct chemicals (DDT, arsenic, copper, nickel, lead, zinc, silver, zinc, arsenic, and selenium) in various freshwater species, considering the dependence of toxicity on temperature. A downward trend in antioxidant enzyme activity in grass carp fish (*Ctenopharyngodon idella*) was related to elevated temperatures combined with mercury exposure, according to the study by Li et al.^7^. Additionally, Moltumyr et al.^4^ investigated the potential welfare consequences of periodic warm water treatments on Atlantic salmon (*Salmo salar*) over an extended period of time. Under 25 °C (control) and 30 °C (experimental), hematological, biochemical, genotoxic, and histopathological indicators were assessed as biomarkers for heat stress in the neotropical catfish (*Rhamdia quelen*)^8^. Klein et al.^9^ conducted a study to investigate the impact of increasing temperatures on the antioxidant defense system and oxidative stress indices in two Antarctic fish species, namely *Notothenia coriiceps* and *Notothenia rossii*. Furthermore, Schleger et al.^10^ conducted an assessment of the antioxidant defenses in the freshwater fish *Astyanax lacustris* under conditions of heat stress.

The black sand is naturally occurring mineral depositions along the coastal areas, dominated by an iron titanates and minor oxides such as calcium, magnesium and silicon^11^. Other strategic and economic minerals like rutile, monazite, leucoxene, magnetite, garnet, and zircon were also present in these deposits. Black sand placer deposits are found in the form of sediments along beaches and coastal sand dunes. Numerous studies in radiometry and mineralogy have been conducted on these sands^12–19^. X-ray Diffraction (XRF) analysis of black sand collected from the Egyptian Red Sea coast revealed the presence of various oxides, such as Iron(III) oxide (Fe_2_O_3_), Silicon dioxide (SiO_2_), Titanium dioxide (TiO_2_), Magnesium oxide (MgO), Calcium oxide (CaO), and Aluminum oxide (Al_2_O_3_)^20^. Studies examining how black sand affects animals in the wild or through experiments are relatively uncommon. The impact of black sand, which was isolated from the beach of the Red Sea, on *Oreochromis niloticus* was assessed by Saad and Sayed^20^. More recently, Abdelbaki et al.^21^ investigated the structure, spreading, and occurrence of black sand along the Red Sea, Egypt, through a comprehensive field and lab investigations.

An expanding class of anthropogenic contaminants, nanoparticles interact with their surroundings in a variety of ways. In numerous industrial fields, including the manufacture of paints, personal care products, biological sensors, and electrical catalysts, NPs are extensively utilized^22^. NPs from industrial or domestic waste can alter the aquatic ecology and endanger aquatic life when they are released into the environment^23^. According to studies, Titanium dioxide nanoparticles (TiO_2_-NPs) may cause DNA damage, oxidative stress, inflammation, genotoxic response, and tissue distribution^24,25^. *C. gariepinus* showed changes in behavior, neurological and physiological markers after being exposed to Silicon dioxide nanoparticles (SiO_2_-NPs) nanoparticles^26^. Also, adult zebrafish (*Danio rerio*) that exposed to SiO_2_-NPs showed impaired reproductive performance and oxidative stress^27^.

It has been indicated that the combined influence of elevated temperatures and chemical pollution synergistically modifies aquatic ecosystems^28^. In this scenario, the consequences of elevated temperatures and the presence of carbon nanotubes on the biochemistry and physiology of *Mytilus galloprovincialis* were studied^29^. Also, Baag et al.^30^ conducted an experimental evaluation to evaluate the combined effects of increased temperature and zinc oxide nanoparticles on the mud crab *Scylla serrata.*

The most significant species of fish used in aquaculture, the African catfish (*Clarias gariepinus*) is widely popular as a source of protein due to its low diseases mortality rate^31^. Utilizing histopathological indicators and evaluating biochemical markers are advantageous techniques for figuring out how animals respond to stressors^32^.

The individual and combined impacts of rising water temperature and BS-NPs on catfish *C. gariepinus* are poorly understood. Accordingly, the current work aimed to study the impact of water temperature increases and BS-NPs separately and in combination on measures of renal function, antioxidant responses, neuro-stress indicators, and histological examinations in *C. gariepinus*.

## Materials and methods

### Sampling of the black sand (BS)

The BS samples were obtained from Hurghada city, the Red Sea beach, Egypt (S.D 1). The isolation, and separation of BS were done according to Saad and Sayed^20^. The nanoparticles were synthesized via top-down approach, in this method the BS-NPs sample was grinded using a ball-milling machine (Fritsch Mini-Mill pulverisette 23, GmbH, Duisburg, Germany) for 1 h.

### Characterization of BS-NPs sample

A comprehensive analysis of the BS-NPs sample was investigated at Assiut University. X-ray fluorescence (XRF) at the Faculty of Engineering revealed their chemical composition. The morphology and particle size of the BS-NPs were examined utilizing transmission electron microscopy (TEM). Images were captured with a JEOL Model JSM-5400 LV (Joel, Tokyo, Japan) set to 80 kV. X-ray diffraction (XRD) using a Phillips PW 2103/00 diffractometer with Ni-filtered CuKα radiation was employed to determine the crystalline phases, nature, and crystal size. Finally, the functional groups present in the samples were identified using Fourier-transform infrared (FTIR) spectroscopy, employing a Nicolet 6700 spectrophotometer.

### Fish collection and exposure conditions

*Clarias gariepinus* of good health, displaying no signs of infection, were procured from a local fish farmer. Catfish were (male and female) had an average weight of 200 ± 25 g and average length of 25 ± 5 cm. The experiments adhered to Test Guideline No. 203 Fish; Acute Toxicity Testing outlined by OECD^33^ with slight modification. Prior to experimentation, catfish were acclimatized in 120 L containers for 15 days under controlled conditions at a temperature of 26 ± 2 °C, pH level range of 7.2–7.6, oxygen saturation at a minimum of 80%, and a natural photoperiod of 12 h of darkness followed by 12 h of light. Twice daily, the *C. gariepinus* were fed a commercial diet (SKRETTING, Egypt) containing 30% protein. The diet included ingredients such as soy meal, wheat bran, maize, crude protein, lipids, crude fibers, fish meal, calcium, and sodium chloride, following the guidelines set by the Organization for Economic Co-operation and Development (OECD) in 2019^33^. Maintaining good water quality, 40% of the water was changed daily to remove fish waste.

### Experimental design

Four groups were randomly selected from a total of 120 *C. gariepinus*. In each group, the samples were kept in glass containers with 120 L of water (30 per group) each group had three replicate tanks were included (10 fish per tank). Group 1, the control group, was given a normal diet at a standard temperature of 26 ± 2 °C, Group 2 received a diet containing 6.4 g NPs/kg of BS-NPs according to Saad and Sayed^20^ at a normal temperature of 26 ± 2 °C. Group 3 received a normal diet with a Heat Stress (HS) of 32 °C according to Khieokhajonkhet et al.^34^. Group 4 received food comprising 6.4 g/kg BS-NPs with a water temperature of 32 °C. Following a 15-day exposure, six fish were randomly chosen from each group, euthanized and immersed in a cold bath ranges between 10 and 15 °C (50°F–59°F) to reduce stress levels^35,36^. Following caudal puncture, blood received in non-heparinized tubes from the caudal vein. serum samples were stored at − 80 °C for succeeding biochemical investigation after being centrifuged for 10 min at 5000 rpm^37^. In the study, serum samples from the fish were analyzed using several assays to assess health and stress responses. Specifically, assays for measuring kidney functions, oxidative stress markers and neuro-stress indicators.

### Kidney functions

According to Hamed et al.^38^, colorimetric measurements of creatinine and uric acid were made utilizing UV/Vis spectrophotometer in the range of 340–546 nm (Biodiagonstic Company, Egypt).

### Antioxidant parameters

The method from Aebi^39^ was used to calculate the level of catalase (CAT). The level of superoxide dismutase (SOD) was evaluated using the method that reported by Nishikimi et al.^40^. The methodology provided by Koracevic et al.^41^ was followed to measure the total antioxidant capacity (TAC).

### Neuro- and stress parameters

To measure serum acetylcholinesterase (AchE) levels, the method outlined by Knedel and Böttger^42^ was employed, utilizing Stanbio kits. Cortisol levels were determined through enzyme-linked immunosorbent assay (ELISA), following the protocol described by Foster and Dunn^43^.

### Histological investigations

Following catfish dissection, kidney and spleen samples were (n = 6 samples) collected from each group and preserved in 10% neutral-buffered formalin. After that, the samples were cleared in xylene and dehydrated using ethanol at increasing concentrations. To prepare the samples for staining, they were first embedded in paraffin wax. Next, the paraffin block was cut into thin slices, 5 µm thick, using a microtome. Finally, these sections were stained with three different histological stains to visualize specific tissue structures. Hematoxylin and Eosin (H&E) for a comprehensive histological examination^44^. A special staining technique called Periodic Acid Schiff (PAS) was used to identify carbohydrates in the tissue^45^. Another technique, Sirius Red staining, was used to visualize collagen fibers^46^. The prepared sections were then checked under a light microscope (Olympus, USA) by a histopathologist who was unaware of the group assignments.

### Statistical analysis

GraphPad Prism 9.0 (GraphPad Software Inc.) was used to statistically analyze all the data, and the results were shown as mean ± standard error of the mean (SEM). To see if there were any important differences between the control group and the other groups, a statistical test called one-way ANOVA was used. Then, another test, Fisher's LSD, to pinpoint exactly which groups were different from the control. Differences between treatments and controls were believed statistically significant at *p* ≤ 0.05. The asterisk superscript (*) was used graphically to indicate the significance level (*p < 0.05, **p < 0.01, ***p < 0.001, ****p < 0.0001).

## Results

### Characterization of BS NPs sample

**Fig. 1:**
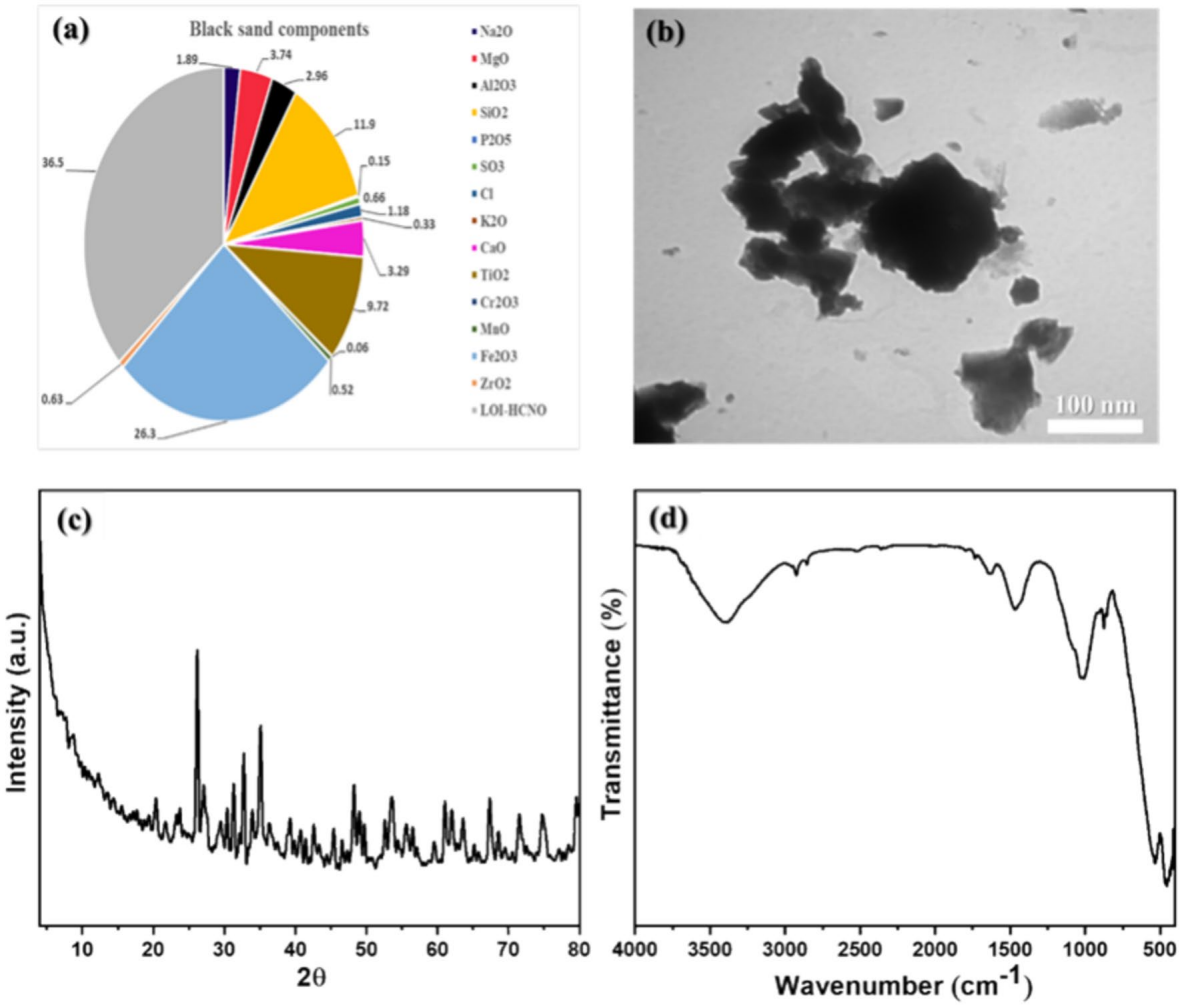
Illustrates the comprehensive characterization of BS-NPs sample. (**a**) Shows the elemental composition of the BS-NPs as determined by X-ray fluorescence (XRF), indicating the presence and abundance of key elements. (**b**) Presents a transmission electron microscopy (TEM) image revealing the morphology and size distribution of the nanoparticles. (**c**) Displays the X-ray diffraction (XRD) pattern, which provides insights into the crystalline structure of the BS-NPs. (**d**) Depicts the Fourier-transform infrared (FTIR) spectrum, identifying the functional groups associated with the nanoparticles.

The semi-quantitative chemical analysis of the BS-NPs sample was conducted by XRF. Analysis of the BS-NPs sample (Fig. 1a) revealed that Fe_2_O_3_ makes up the largest portion (26.3%) followed by SiO_2_ at 11.9%. TiO_2_, MgO, CaO, and Al_2_O_3_ were also present in smaller amounts (around 3–10%). The remaining metal oxides were each less than 2% of the sample. Figure 1b shows the TEM micrograph of the dispersion of BS-NPs sample in ethanol deposited over a Cu grid. The BS-NPs sample consists of a dispersion of almost plate-like particles with an average particle size of 266 nm. The black color particles are attributed to a magnetite particle, while the gray particles are related to quartz. The XRD analysis of the BS-NPs sample is depicted in Fig. 1c. It shows the BS-NPs sample is crystalline and has numerous diffraction peaks, the peaks at 2*ϴ* values of 20.4°, 26.2°, 41.4° and 49.7° are attributed to high SiO_2_ based on the standard card (JCPDS No. 00-011-0252). The detected peak at 59.5° may be related to SiO_2_ (JCPDS No. 00-003-0393), and the peaks at 30.4°, 31.3°, 33.9°, 39.2° and 63.6° may be due to Albite, Al_2_O_3_⋅Na_2_O⋅6SiO_2_ according to the standard card (JCPDS No. 00-002-0515). The two peaks at 42.6° and 61° are due to Mullite 3Al_2_O_3_⋅2SiO_2_ (JCPDS No. 00-002-1160) and the peaks at 29.4°, 35.1°, 36.3°, 53.5°, 62° and 74.5° have belonged to Fe_2.932_O_4_ (JCPDS No. 01-086-1352). In addition, the two signals at 32.7° and 67.4° are due to Fe_2_O_3_ (JCPDS No. 00-016-0653), while the peak at 33.9° is related to AlFeO_3_ (JCPDS No. 00-030-0024). Moreover, the two reflections at 46.5° and 52.6° are ascribed to FeCO_3_ (JCPDS No. 00-012-0531), whereas at 45.4° is assigned to iron Fe_2_Ti_4_O (JCPDS No. 01-075-0402). Furthermore, the signals at 23.7°, 40.7°, 48.2°, 56.5°, 71.5° and 79.5° are recognized to Ti_2_O_3_ (JCPDS No. 01-071-1056), and at 39.2° has belonged to TiO_2_ (JCPDS No. 00-034-0180). The observed peaks at 27.1°, 49° and 55.5° are ascribed to CaCO_3_ (JCPDS No. 01-074-1867). Figure 1d shows the BS NPs sample's FTIR spectrum. The O–H stretching and bending modes of hydroxyl groups are responsible for the bands seen at 3397 and 1633 cm^−1^, respectively. Furthermore, the symmetric and asymmetric vibrations of the C–H of saturated aliphatic compounds are responsible for the bands that were identified at 2926 and 2855 cm^−1^, respectively. Asymmetric stretching of C–O, stretching vibrations of Si–O–Si, Fe–O bond, Fe–O stretching, Fe–O stretching or Ti–O bond vibration, and Si–O bending are the explanations given for the bands found at 1472, 1008, 874, 534, 456, and 418 cm^−1^, respectively.

### Kidney functions

**Fig. 2:**
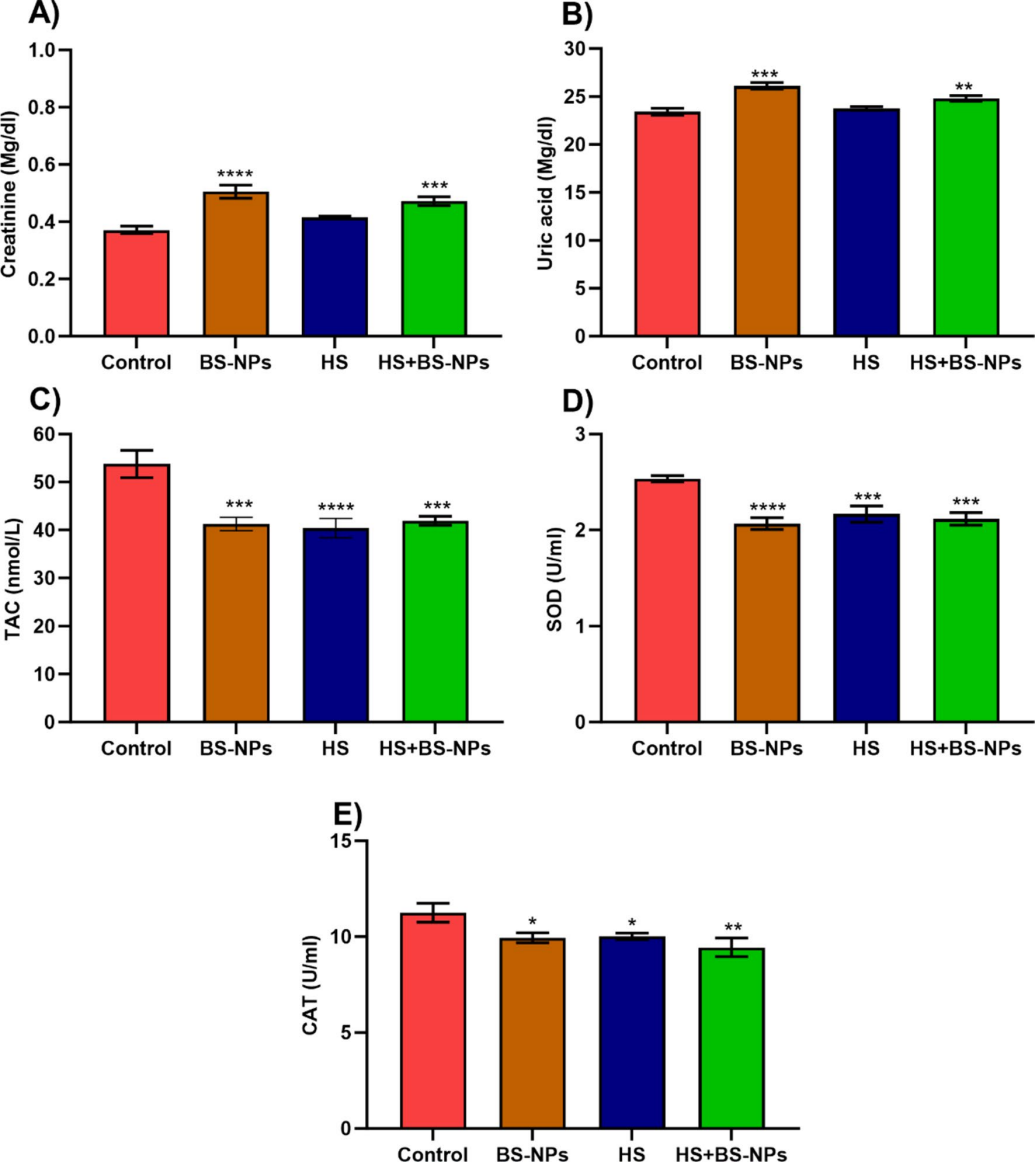
Illustrates the impact of BS-NPs and heat stress on kidney functions and oxidative response parameters of *Clarias gariepinus*. (**A**) shows the levels of creatinine in the kidney, (**B**) the concentration of uric acid, (**C**) total antioxidant capacity (TAC), (**D**) superoxide dismutase (SOD) activity, and (**E**) catalase (CAT) activity. Data are presented as mean ± SEM with statistical significance indicated by *p < 0.05, **p < 0.01, ***p < 0.001, ****p < 0.0001.

The resulting data of kidney function measured in the serum of *C. gariepinus* are graphically presented as means ± SEM (Fig. 2A,B). Compared to control fish; the mean value of creatinine scored from BS-NPs and HS + BS-NPs was significantly higher (*p* < 0.001). Even though the mean creatine value measured in HS exposed group was higher than the control one, it was not statistically significant. As well as creatinine, the mean value of uric acid measure in BS-NPs and HS + BS-NPs was higher (*p* < 0.001) compared to the control. Uric acid measurements in the HS group were very close to control values (*p* > 0.05).

### Antioxidant parameters

The effects of BS-NPs and HS on the antioxidant indicators (TAC, SOD, and CAT) in *Clarias gariepinus* is shown in Fig. 2 (0.05 ≥ *p* < 0.0001). TAC was recorded from the BS-NPs, HS, and their combination (HS + BS-NPs) showed the highest levels of significance compared to the control group. Similarly, the average SOD data from each treatment showed a comparable decline trend when compared to the control group (*p* < 0.001) (Fig. 2C). On the other hand, the heat stress had a greater impact on the mean TAC than SOD (Fig. 2D,E), with the latter showing a higher BS-NPs induced drop.

### Neurological parameters

**Fig. 3:**
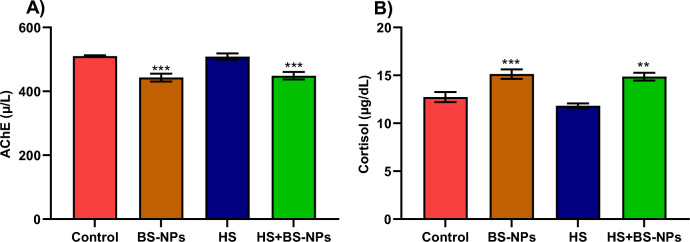
Illustrates the impact of BS-NPs and heat stress on neurophysiological parameters of *Clarias gariepinus*. (**A**) Shows the activity of acetylcholinesterase (AchE), and (**B**) depicts the levels of cortisol. Data are presented as mean ± SEM with statistical significance indicated by **p < 0.01 and ***p < 0.001.

The AchE activity in the HS group was not significantly different from the control group (*p* > 0.05). In contrast, the AchE activity in the BS-NPs group and the HS + BS-NPs group was significantly different from the control group (*p* < 0.001), indicating a distinct divergence from the control values (Fig. 3). Conversely, cortisol levels rose in both the BS-NPs and HS + BS-NPs groups relative to the control fish. Nevertheless, there was no statistically significant difference in the mean cortisol levels between the HS and the control group (*p* > 0.05).

### Histopathological and histochemical studies

#### Kidney

**Fig. 4:**
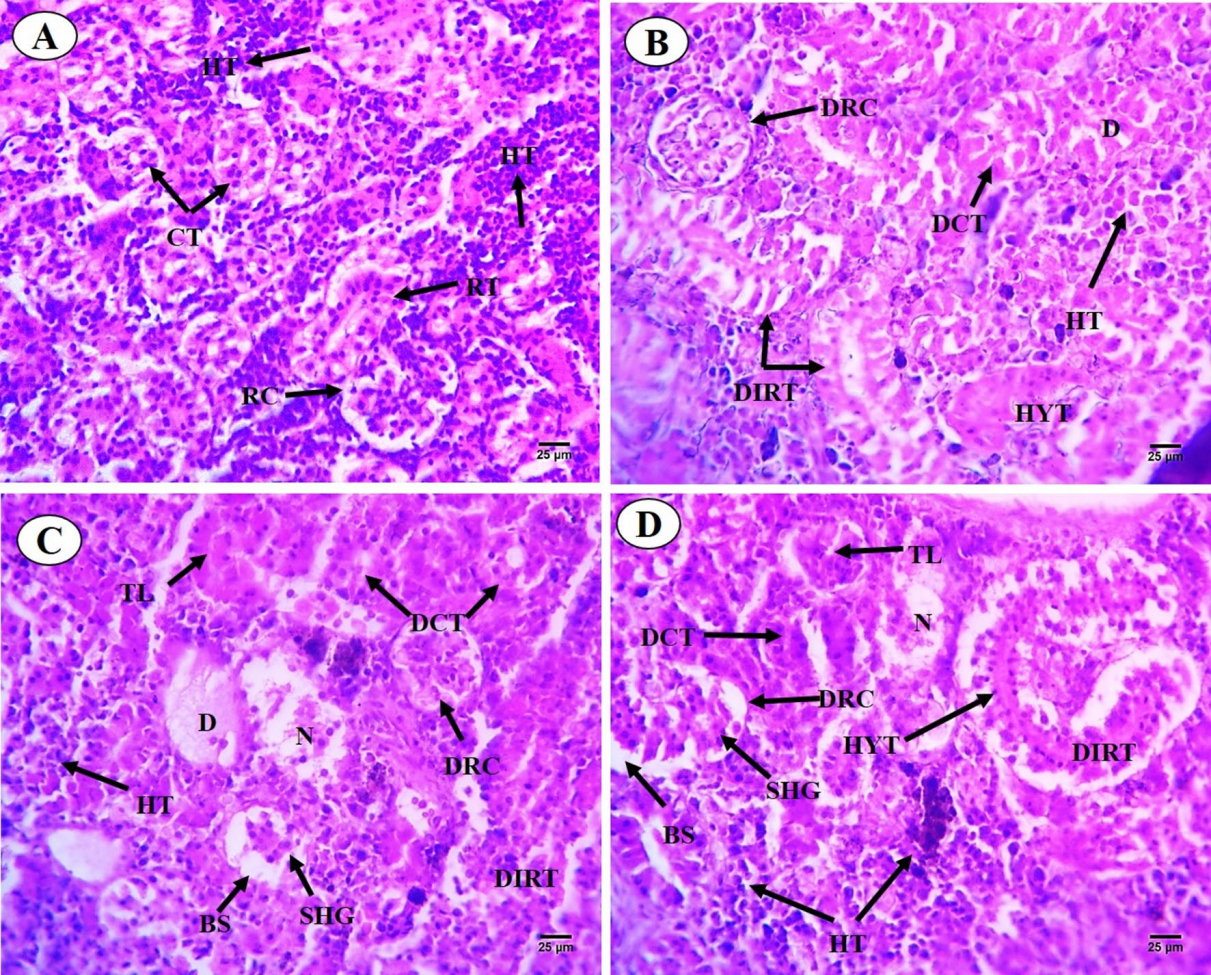
Transverse sections of control and treated fish kidney (H&E X 400). (**A**) Sections of control fish kidney showing the renal corpuscle (RC), renal tubules (RT), collecting tubules (CT) and haemopoietic tissue (HT). (**B**) Sections of fish kidney exposure to thermal stress (32C) showing dissociation in renal tubules (DIRT), Hypertrophy in the glomerulus (HYT), degeneration in collecting tubules (DCT) and degeneration in renal corpuscle (DRC). (**C**) Sections of fish kidney exposure to BS nanoparticles (9.6 g BS/kg diet) showing necrosis (N) and degeneration (D), shrinkage of the tubular lumen (TL), shrinkage of glomerulus (SHG) and increasing in the Bowman's space (BS). (**D**) Sections of fish kidney exposure to combination of both treatments showing dissociation (DIRT) and hypertrophy (HYT) in the renal tubules, shrinkage of the tubular lumen (TL), degeneration in the renal corpuscle (D), shrinkage of glomerulus (SHG) and increasing in the Bowman's space (BS).

**Fig. 5:**
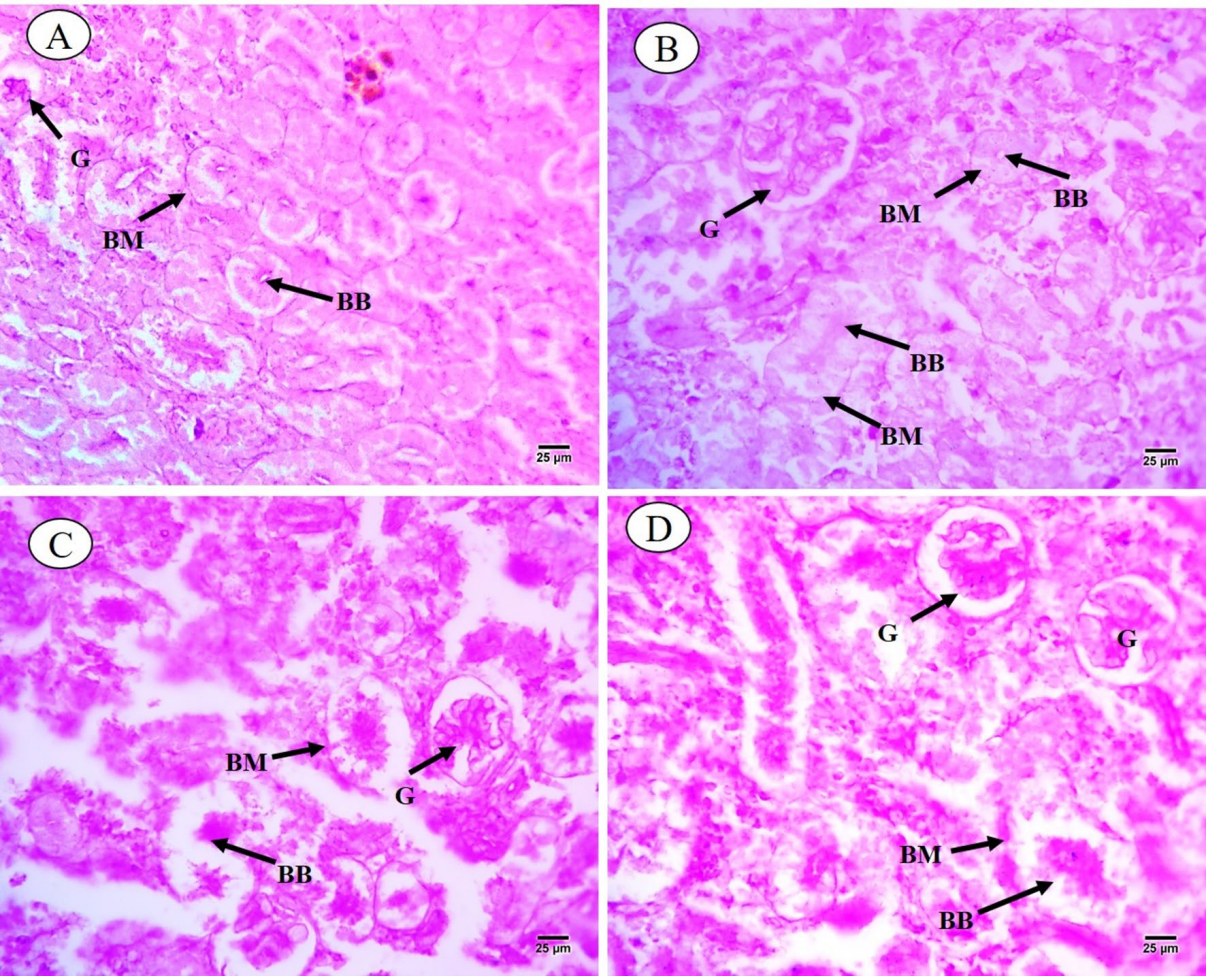
Transverse sections of control and treated fish kidney (PAS X 400). (**A**) Sections of control fish kidney showing positive PAS reactivity in brush border (BB) and the basement membrane (BM) of the renal tubules. (**B**) Sections of fish kidney exposure to thermal stress showing moderate decline in polysaccharide materials in brush border (BB), the basement membrane of the renal tubules (BM) and glomerulus (G). (**C**) Sections of fish kidney exposure to BS nanoparticles showing more depletion in polysaccharide materials in brush border (BB), the basement membrane (BM) of the renal tubules, glomerulus (G) and hemopoietic tissue (HT). (**D**) Sections of fish kidney exposure to combination of both treatments showing great depletion in the polysaccharide materials in compared to those of control ones and other treatments.

**Fig. 6:**
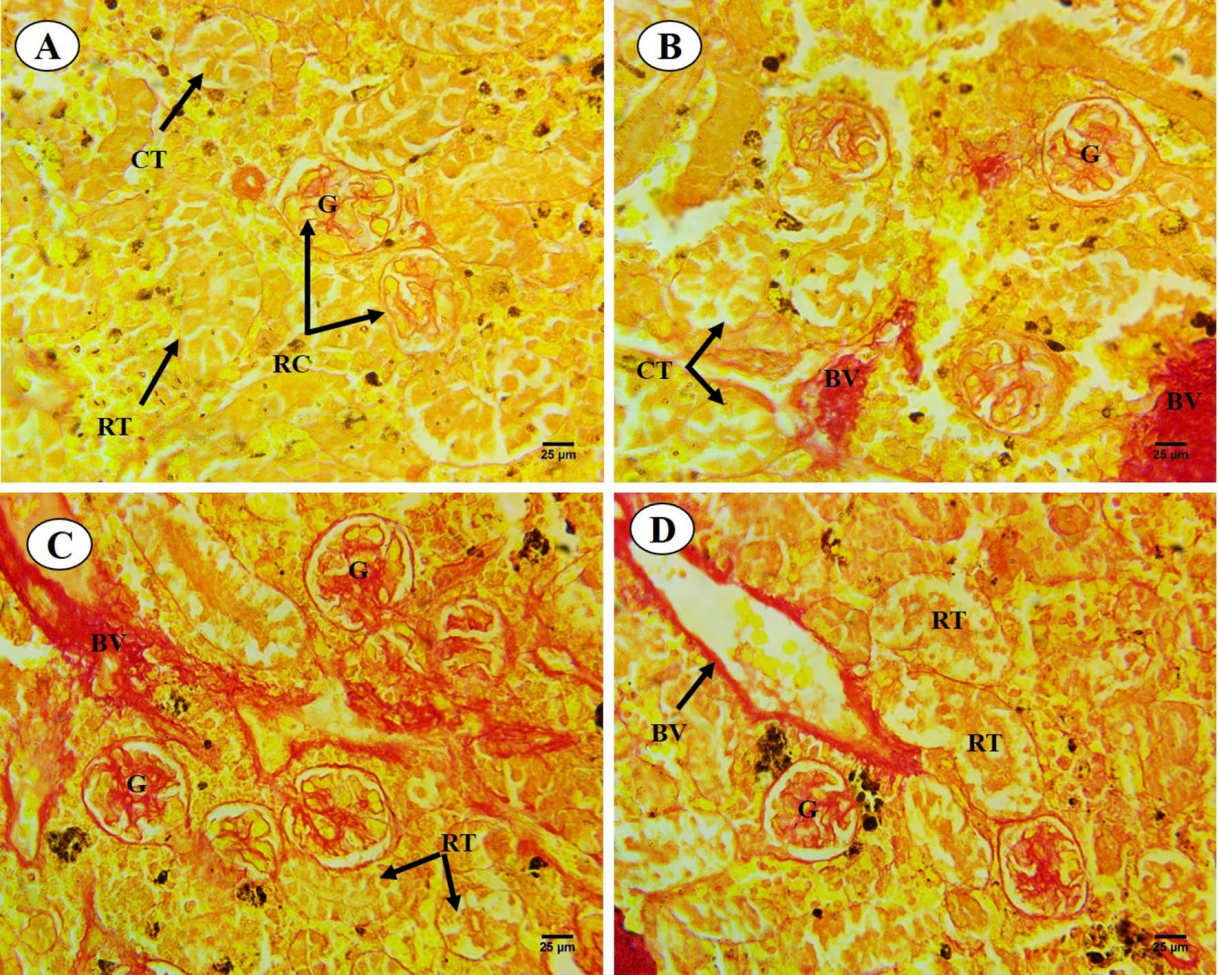
Sections of control and treated fish kidney (Sirius X 400). (**A**) Sections of control fish kidney showing normal distribution of collagen in renal capsule (RC) and the wall of blood vessels (BV). (**B**) Sections of fish kidney exposed to thermal stress revealed density of staining were increased around blood vessels and slightly faint in renal capsule. (**C**) Sections of fish kidney exposed to BS nanoparticles showing increased collagen intensity of staining in blood vessels, glomerulus, and renal corpuscle. (**D**) Sections of fish kidney exposed to combination of both treatments showing more collagen fiber around blood vessels and glomerulus.

Results of histological analysis of the kidney of *C. gariepinus* exposed to thermal stress (32 °C), BS-NPs (9.6 g BS/kg diet), and a combination of both treatments for 15 days are presented in Fig. 4A–D. The kidneys in the control group have normal positions for glomeruli, Bowman's capsules, tubular lumen, blood arteries, hemopoietic tissue, and intra-renal spaces (Fig. 4A). Different degrees of damage, such as dissociation in the renal tubules, hypertrophy and degeneration in the glomerulus, degeneration in the collecting tubules, and proliferation in the hemopoietic tissue, have been seen in fish exposed to heat stress (Fig. 4B). Moreover**,** BS-NPs group revealed adverse impacts on the kidney including various degrees of injuries ranging from dissociation in the renal tubules, shrinkage of the tubular lumen, shrinkage of glomerulus and increasing in the Bowman's space, proliferation in the haemopoietic tissue and degeneration and necrosis of renal tubules (Fig. 4C). The combined treatments caused significant kidney damage, including disrupted architecture, tubular hypertrophy, and glomerular shrinkage, along with increased Bowman's space and necrosis of collecting tubules (Fig. 4D). PAS-technique exhibited positive reactivity in brush border, the basement membrane of the renal tubules and the glomerulus of the control fish (Fig. 5A). Thermal stress-exposed fish revealed moderate decline in polysaccharide materials in brush border, the basement membrane of the renal tubules, and glomerulus (Fig. 5B). PAS-technique in BS-NPs exposed fish revealed more depletion in polysaccharide materials in brush border, the basement membrane of the renal tubules, glomerulus and hemopoietic tissue compared to those of control ones (Fig. 5C). Fish exposure to a combination of both treatments, showing great depletion in the polysaccharide materials in comparison to those of control ones and the other treatments (Fig. 5D). Sections of kidney in all experimental groups stained with Sirius red stain for demonstration of connective tissues collagenous fiber. The control group showed normal distribution of collagen in renal capsule and the wall of blood vessels (Fig. 6A). Thermal stress-exposed fish revealed this density of staining were increased around blood vessels and slightly faint in renal capsule when compared with the control (Fig. 6B). BS-NPs exposed fish displayed increased collagen intensity of staining in blood vessels, glomerulus and renal corpuscle as compared with the control and the thermal stress group (Fig. 6C). Fish exposure to a combination of both treatments revealed more collagen fiber around blood vessels and glomerulus compared to those of the control ones and the other treatments (Fig. 6D).

#### Spleen

**Fig. 7:**
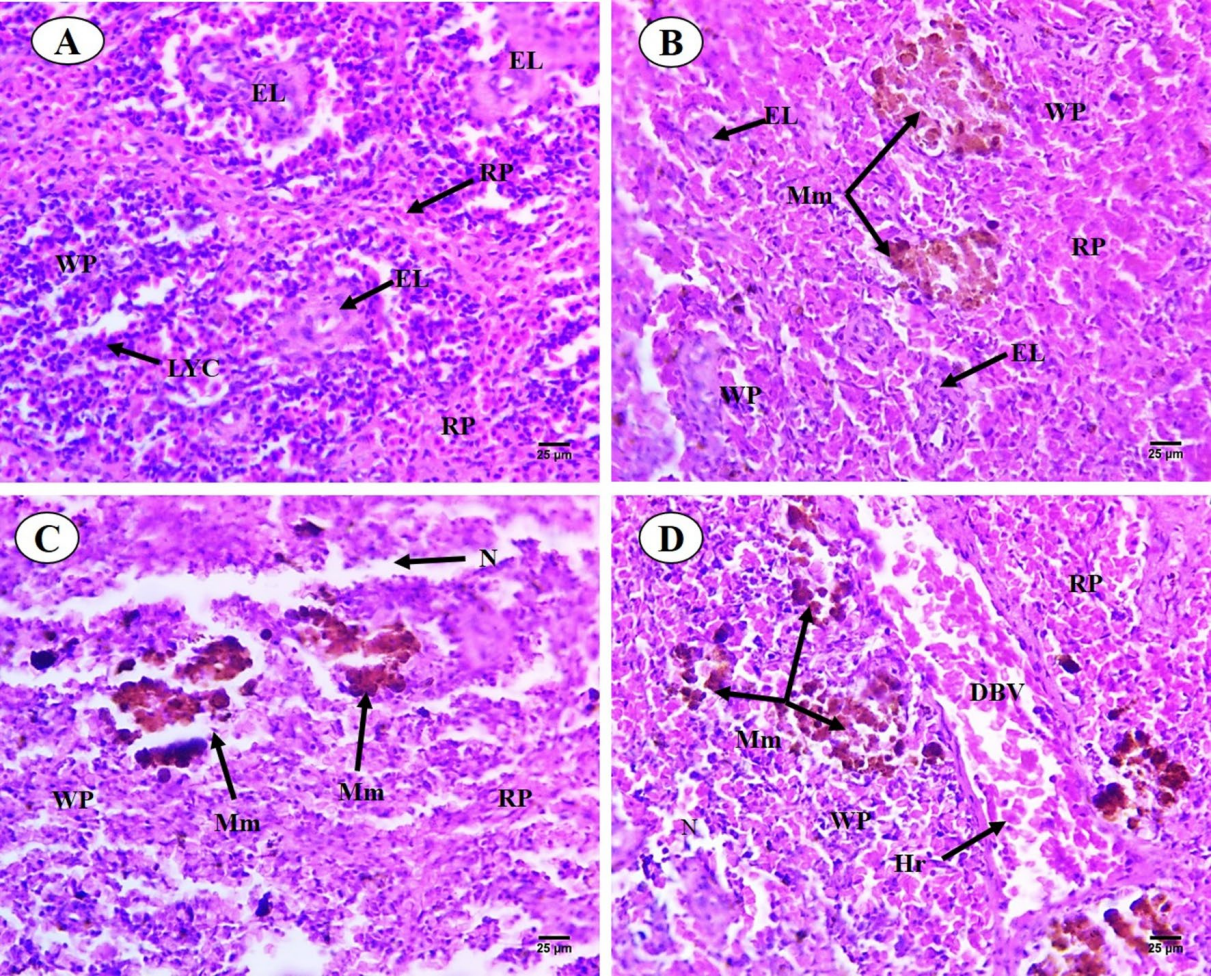
Sections of control and treated fish spleen (H&E X 400). (**A**) Sections of control fish spleen showing red (RP) and white pulps (WP), ellipsoid shape (Es) and lymphocyte (LYC). (**B**) Sections of fish spleen exposed to thermal stress (32C) showing increased in size in white pulp (WP), expansion of the melanomacrophage centers (Mm). (**C**) Sections of fish spleen exposed to BS NPs (9.6 g BS/kg diet) showing necrosis (N) in the white pulp. (**D**) Sections of fish spleen exposed to combination of both treatments showing necrosis (N) in white and red pulps, dilatation of blood vessel (DBV) and hemorrhage (Hr).

**Fig. 8:**
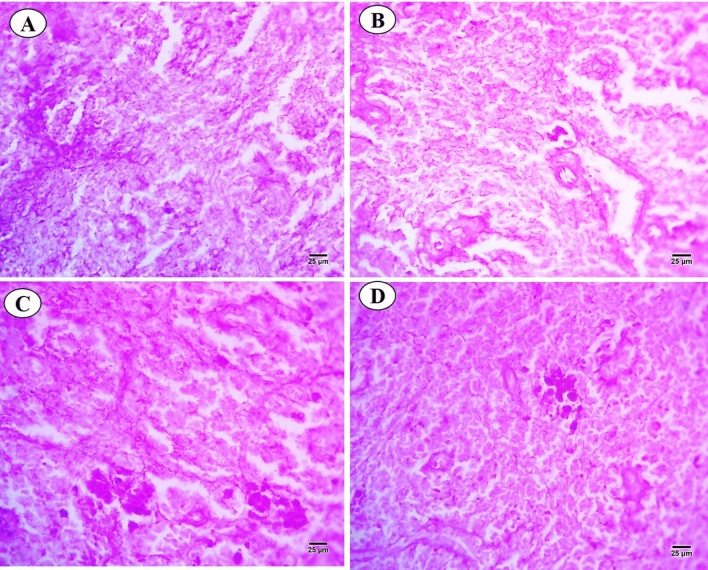
Transverse sections of control and treated fish spleen (PAS X 400). (**A**) Sections of control fish spleen exhibited positive reactivity in white and red pulps and ellipsoid structure. (**B**) Sections of fish spleen exposed to thermal stress showing moderate decline in polysaccharide materials in both white and red pulps also ellipsoid structure. (**C**) Sections of fish spleen exposed to BS nanoparticles showing more depletion in polysaccharide materials in splenic tissues. (**D**) Sections of fish spleen exposed to combination of both treatments showing great depletion in the polysaccharide materials.

**Fig. 9:**
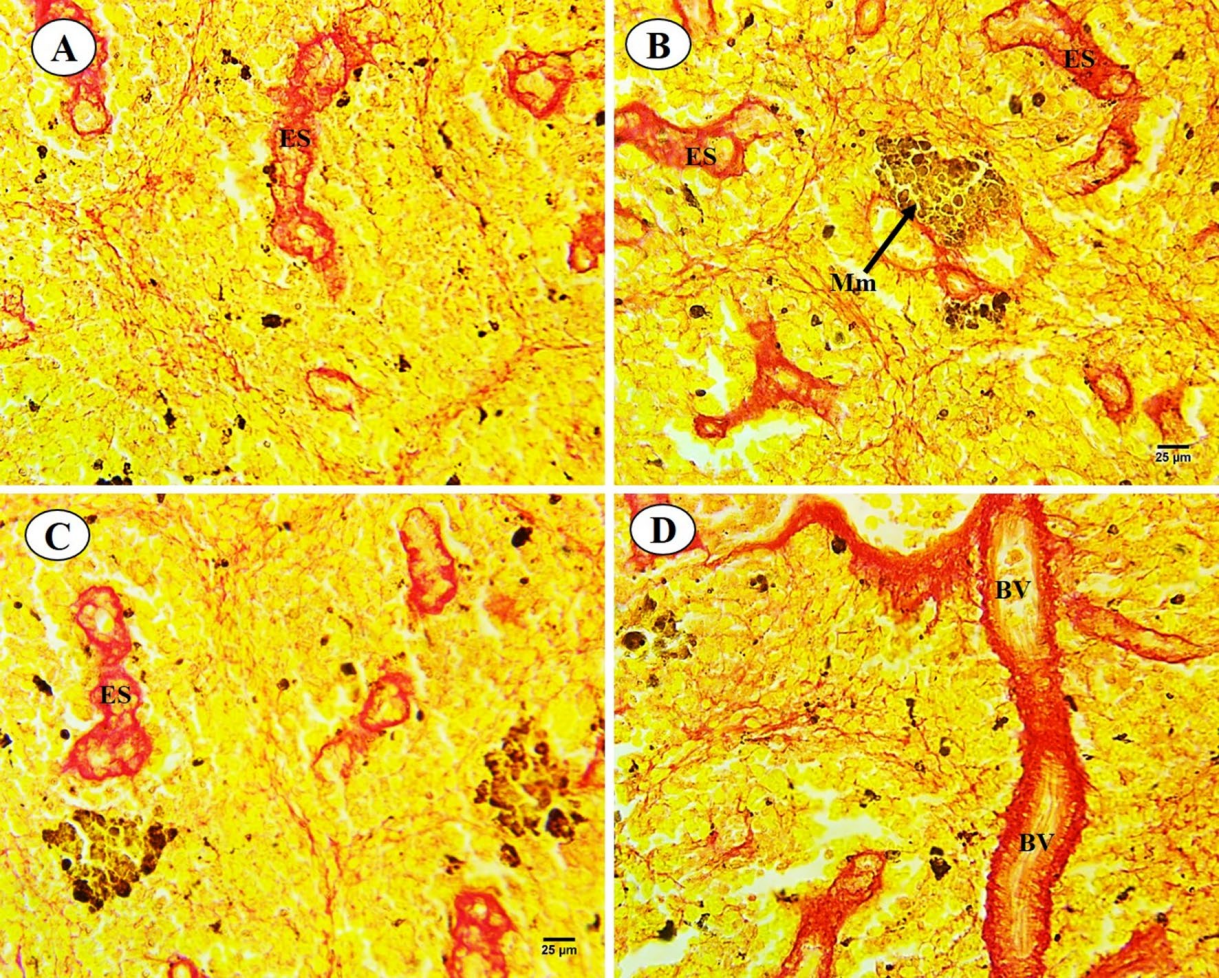
Sections of control and treated fish spleen (Sirius X 400). (**A**) Sections of control fish spleen showing normal distribution of collagen in lining of ellipsoid structure and blood vessels. (**B**, **C**) Sections of fish spleen exposed to thermal stress and black sand nanoparticles showing increased density of staining around blood vessels and in lining of ellipsoid structure. (**D**) Sections of fish spleen exposed to combination of both treatments showing more collagen fiber around blood vessels and lining of ellipsoid structure.

Sections of *C. gariepinus* spleen stained with control and exposed to thermal stress (32 °C), BS-NPs (6.4 g BS/kg diet), and a combination of both treatments are presented in Fig. 7A–D. The control group showed morphology of spleen represented in both red and white pulps were clearly observed, increase in both lymphocytes aggregated in the white pulp and ellipsoid bodies which located in the core of the white pulp. While red pulp full of hemopoietic tissues, especially red blood cells (RBCs) (Fig. 7A). In contrast, there were a variety of alterations in the thermal stress group, including an increase in the size of the white pulp that contained newly produced lymphocytes and an unclear separation between the white and the red pulps. However, expansion of the melanomacrophage centers with large granular, irregular, and brown pigments (hemosiderin). Deformation in the lining (cubic epithelium) of ellipsoid structures which presented as terminal capillaries in both white and red pulps which surrounded by a sheath of acidophilic fibrous connective tissues and dispersed lymphocytes. Shrunken red pulp which contains dispersed hematopoietic tissues (Fig. 7B). In BS-NPs group, red pulp increased in size containing hemopoietic tissues mainly RBCs. The boundary between white and red pulps were not clear in comparison with the control group. Shrinkage and necrosis in the white pulp containing a few newly formed lymphocytes. Melanomacrophage centers with large, irregular, black and brown pigments (melanin and hemosiderin) were noticed. Ellipsoid structures were not observed in both white and red pulps (Fig. 7C). Combined group showed degeneration and necrosis in white and red pulps and dispersed lymphocytes. Boundary between red and white pulps were not observed, also increased in size and number of the melanomacrophage centers with large, irregular, black and brown pigments (melanin and hemosiderin). Vascular dilatation of blood vessels full of blood corpuscles and ellipsoid structures were not observed in both white and red pulps (Fig. 7D). PAS-technique for all experimental groups is shown in Fig. 8A–D. In the control group, positive reactivity in white and red pulps and ellipsoid structure were detected (Fig. 8A). Thermal stress-exposed fish revealed moderate decline in polysaccharide materials in both white, red pulps and ellipsoid structure (Fig. 8B). PAS-technique in the BS-NPs exposed fish revealed more depletion in polysaccharide materials in splenic tissues as compared to those of control ones (Fig. 8C). Fish exposure to a combination of both treatments, showing great depletion in the polysaccharide materials compared to those of control ones and other treatments (Fig. 8D). Sections of spleen in all experimental groups stained with Sirius red stain for demonstration of connective tissues collagenous fiber (Fig. 9A–D). In the control group showed normal distribution of the collagen in lining of ellipsoid structure and blood vessels (Fig. 9A). Thermal stress and BS-NPs exposed fish revealed increased density of staining around blood vessels and in lining of ellipsoid structure as compared with the control group (Fig. 9B,C). While in the combined group, revealed more collagen fiber around blood vessels and lining of ellipsoid structure compared to the control and the other treatments (Fig. 9D).

## Discussion

Fe_2_O_3_ (26.3%), SiO_2_ (11.9%), TiO_2_ (9.72%), MgO (3.74%), CaO (3.29%), and Al_2_O_3_ (2.96%) are the principal oxides identified in the BS-NPs sample. whereas the remaining metal oxides that have been found have levels of less than 2%. This result is consistent with that obtained by Saad and Sayed^20^. The TEM micrograph of the BS-NPs showed plate-like particles with an average particle size of 266 nm. The black color particles are attributed to a magnetite particle, while the gray particles are related to quartz as stated by Li et al.^47^ and Prasdiantika et al.^48^. The XRD pattern of the BS-NPs sample showed several diffraction peaks. The analysis confirms that the main components of this sample are iron compounds, along with silicate (containing silicon), titanate (containing titanium), and calcite (calcium carbonate)^49^. This composition is consistent with the results obtained from the XRF analysis earlier. The average crystallite size for the BS-NPs sample was evaluated utilizing Scherrer equation^21,50–52^ and found to be equal to 18.6 nm. The O–H stretching and bending modes of hydroxyl groups are responsible for the bands seen in the FTIR spectrum of the BS-NPs sample at 3397 and 1633 cm^−1^, respectively^53^. Furthermore, the symmetric and asymmetric vibrations of the C–H of saturated aliphatic compounds are responsible for the bands that were identified at 2926 and 2855 cm^-1^, respectively. Asymmetric stretching of C–O, stretching vibrations of Si–O–Si, Fe–O bond, Fe–O stretching, Fe–O stretching or Ti–O bond vibration, and Si–O bending are the explanations given for the bands found at 1472, 1008, 874, 534, 456, and 418 cm^-1^, respectively. Knowing the content of BS-NPs is crucial for accurately interpreting their biological effects, assessing safety, and understanding the mechanisms through which they interact with biological tissues^49^. The elemental composition and structural properties of these nanoparticles determine how they behave in biological systems, influence their toxicity, and provide insights into their potential applications^54^.

Diseases like lung cancer, pancreatic, liver, and kidney have been associated with a variety of sources of radioactive materials released from the terrestrial radionuclides ^238^U, ^226^Ra, ^232^Th, and ^40^K, as well as radon gas and its daughters and ingesting radioactive food^55,56^. Radioactive BS was discovered in the Rosetta coastal zone during an aerial gamma ray spectrometry investigation carried out in 2003 by the Nuclear Material Authority of Egypt^19^. The main source of radioactive enrichment^57^ was the monazite-rich BS beach on Penang Island, Malaysia. Beach sand samples collected on the Mediterranean coast of Egypt, specifically from Baltim beaches in Kafr El-Sheikh governorate, were found to contain naturally occurring radioactive isotopes, including ^226^Ra, ^232^Th, and ^40^K^16^.

Studies comparing different sands for solar stills have shown that BS absorbs heat much better than yellow sand, making it more efficient^58^. Similarly, research by Omara and Kabeel^59^ found black sand to be the most efficient heat absorber among various sands tested in solar stills. This suggests that BS is probably going to contribute to warming and heat stress in nearby aquatic and terrestrial environments.

The long-term effects of human-caused climate change have drawn more attention to the implications of temperature variation for ecology and evolution. Regarding temperature-dependent chemical toxicity in freshwater fish, research has shown that the median lethal concentration (LC_**50**_) for ten chemicals exhibits an inverted V-shaped distribution along a temperature gradient^6^. Fish lack the ability to regulate their body temperature, making them susceptible to temperature increases^60^. Consistent to this, male sexual characteristics appear to be more susceptible to continuous warming, as evidenced by the shorter length and slower growth of *Poecilia reticulata* sperm exposed to temperature fluctuations^5^. Thermal delousing, which uses heated water to remove salmon lice, has grown in the salmon industry since 2015^61^. There's a significant knowledge gap regarding the impact of thermal delousing on salmon health and welfare. Additionally, the long-term consequences after treatment have not been extensively studied^62^. Investigating internal tissues and other indicators after stress is therefore crucial. For example, kidney was identified as a critical accumulator tissue because of their great accumulative potential towards different NPs^63^. The present study has been supported by previous investigations that evaluated an elevation in kidney biomarkers following exposure to heat stress and nanomaterials. Similar to the current study, Dawood et al.^64^ reported a significant effect of heat stress (32 °C) on *C. gariepinus* urea and creatinine. The plasma concentrations of creatinine and uric acid in *O. niloticus* increased significantly upon exposure to aluminum oxide nanoparticles^65^. Ali et al.^66^ revealed elevation in urea and creatinine *Rattus norvegicus* exposed to silica-based nanoparticles. Alterations in renal blood flow, renal function, or production of urine can all contribute to a rise in creatinine^67^ and the histological changes in kidneys that are currently being detected may support this. *C. gariepinus* and *O. niloticus* showed higher urea levels after being exposed to atrazine, mancozeb, chlorpyrifos, lambda-cyhalothrin, and their combination^68^. The current findings agree with Mahmoud et al.^69^, they concluded that the concentrations of creatinine and uric acid exhibited different significant alteration in *C. gariepinus* treated with silver NPs. Together, the increased levels of creatinine and uric acid as well as the histological deterioration imply that BS and heat may be detrimental to catfish.

Furthermore, in the current research, temperature had a significant impact on the antioxidant parameters both alone and in conjunction with BS NPs, demonstrating the combined effects of thermal stress. Consistent with our findings, research investigating the impact of nanomaterials on fish antioxidants has revealed a downward trend in the antioxidant enzyme levels SOD, CAT and TAC. The current findings may corroborate recently published research showing that temperature fluctuations and photoperiod variations have a significant effect on the activity of antioxidant enzymes in Atlantic salmon^70^. The reason antioxidants respond to heat stress is most likely due to the fact that, at a certain temperature, cellular metabolic activity increases until it reaches a threshold, beyond which the metabolic rate decreases^71^. Also, under heat stress, substantial changes in serum, lysozyme, aminotransferase, liver SOD, GPX, and malondialdehyde levels were resulted in salmon^72^. Because they catalyzed the superoxide dismutation reaction, SOD are considered essential components of the first stage of the enzymatic antioxidative defense mechanism^73^. Similarly, Waheed et al.^74^ observed a noteworthy reduction in antioxidant enzymes SOD and CAT, in *O. niloticus* subjected to mercury chloride combined with heat stress.

In addition to kidney and oxidative biomarkers, cortisol is released in response to stress, which alters the neuroendocrine system and causes a number of physiological and metabolic processes that facilitate the tissues' easy absorption of glucose and lactate^75^. Moreover, extreme suppression of AchE activity disrupts nervous system function since AchE can provide important information about neuronal activities essential for survival and performance^76^. In accordance, AchE activities in *Mytilus galloprovincialis*, showed significant changes in behavior and oxidative status following the exposure to nanoparticles and phosphonates^22^. Likewise, *D. rerio* subjected to SiO_2_-NPs showed notable deficits in the AchE and the cortisol^27^.

Histologically, triple-stained sections of the kidney and spleen employed in this work demonstrated the combined effects of heat and BS stress^77^. The present work, along with histopathological investigations carried out by other authors, has yielded a beneficial approach such neurological and oxidative stress for assessing the adverse effects related to heat stress nanomaterials^78^. The BS NPs-induced histopathological alterations observed here agree with previous works conducted on different stressors and vertebrate species including fish^78^. For example, in catfish *Mystus vittatus* exposed to ZnS NPs^79^, *C. gariepinus* exposes to silver NPs^80^, *Rattus norvegicus* exposed to silica NPs^81^ polystyrene NPs-exposed *Hypophthalmichthys molitrix*^82^. Comparable to the current findings Monfared et al.^83^ reported shrinkage in glomerular diameter and formation of intra cytoplasmic vacuoles in the kidney of *Oncorhynchus mykiss* subjected to silver NPs. Copper NPs was reported to had an impact on the histological structure of liver and kidney in *Cyprinus carpio*^84^.

TiO_2_NPs increased gill histopathological damages in *C. carpio* when combined with Ag NPs^85^. This demonstrates the detrimental effect of TiO_2_ and others (e.g., SiO_2_) analyzed in the current investigation and supports our suggestion that BS NPs are deposited in tissues. Supporting study^86^ has demonstrated that *O. niloticus* exhibited tissue (ranked as: spleen > intestine > kidney > liver > gills > brain > muscles) accumulation following exposure to Fe_2_O_3_ NPs. TiO_2_ NPs have been demonstrated to be immunotoxic to fish and to lessen fish neutrophils capacity against bacteria^87^. In aquatic environments variations in temperature and acidity affect the toxicity and bioavailability of some pollutants, such heavy metals and alter the biological processes. The amplified negative chemical effects brought on by the high temperature are probably linked to the disruption in oxygen consumption and aerobic scope^88^.

Histopathological changes in the gills, such as aneurysm hemorrhage, epithelial cell hypertrophy, hyperplasia, and skin ulcers, were linked to the treatment against salmon lice, which involved raising the temperature of the farmed fish to 34 °C for 30 s^62^. In line with the present investigation, Vicentini et al.^8^ found that catfish *R. quelen* displayed a number of sublethal changes at temperature 30 °C, including changes to their hematological system, activation of their antioxidant system, and modifications to their energy metabolism.

Interestingly, changes in kidney function and oxidation indicators, imply that the kidney and spleen degenerative lesions are most likely mediated by the oxidative stress brought on by the tissues-deposited NPs^24^. These combined changes appear to indicate the production of peroxides, free radicals, and reactive oxygen species (ROS), which are linked to cellular damage and lipid peroxidation^89^. In addition to kidney deterioration, the current study also showed several splenic degenerations, including necrotic white and red pulps, hemorrhage blood vessel dilatation, a decrease in polysaccharide materials, aggregated collagen fiber around blood vessels, and ellipsoid structure lining. These findings are consistent with earlier research on stressors and spleen-mediated toxicities. For example, *O. niloticus* exposed to iron oxide NPs^86^, *C. gariepinus* exposed to Thiamethoxam^90^, seabream *Sparus aurata* exposed to zinc oxide particles^91^, carp exposed to Avermectin^92^, zebrafish exposed to acrylamide^93^ and (Di(2-ethylhexyl) phthalate)^94^. The occurrence of oxidative stress, inflammation, and apoptosis is typically strongly linked to splenic injury^92^.

Consequently, even though there are many applications for nanoparticles, their chemical stability and ability to exist on a variety of surfaces and forms helps amplifies their presence and transformation in ecosystems^95^. According to the current findings, along with previous works, temperature and chemical exposure have a complex mixed effect on aquatic species. Our histology findings on catfish are consistent with those of Saad and Sayed^20^ on *O. niloticus* subjected to 15 days of BS. Their findings indicated that the liver, intestines, and brain exhibit various alterations including cellular degeneration, vacuolation, infiltration, hemorrhage congested blood vessels, etc.

The study findings of Abdelbaki et al.^21^ provided useful knowledges into the geological diversity of BS occurrences along the Red Sea, they underlined the need for additional research and evaluation. In accordance, the current study provided a sizable dataset about the ecotoxicological effects and potential impact of black sand on the aquatic ecosystems and associated fauna.

Future directions could involve studying the long-term and chronic effects of BS-NPs on fishes, exploring different concentrations and environmental interactions, and using advanced molecular techniques for deeper mechanistic insights. Expanding the range of biomarkers and refining the nanoparticle synthesis process could also enhance the study's relevance and applicability in real-world scenarios.

## Conclusion

The heat stress and BS-NPs caused different degrees of damage to the kidney and spleen, in addition to altered kidney, oxidative, and neurological signs. According to the chemical structure, BS contains a few substances that have been linked to the harm of aquatic life and associated organisms. These findings suggest that, in addition to the changes in temperature brought on by global warming, BS presents an increasing risk to the adjacent aquatic and terrestrial environments. The combined stresses of pollution exposure and climate change may be detrimental for biota that are already on the border of their physiological tolerance limit. Accordingly, further research is still required to make sure that nanomaterials will not have any harmful effects before we use them in industrial household products and public health-related settings.

## Data Availability

All relevant raw data will be freely available from the corresponding author.

## References

[1] Maulvault, A. L. *et al.* Ecophysiological responses of juvenile seabass (*Dicentrarchus labrax*) exposed to increased temperature and dietary methylmercury. *Sci. Total Environ.* **586**, 551–558. 10.1016/j.scitotenv.2017.02.016 (2017). 28216029 10.1016/j.scitotenv.2017.02.016

[2] Woodward, A. *et al.* Climate change and health: On the latest IPCC report. *Lancet* **383**, 1185–1189 (2014). 24703554 10.1016/S0140-6736(14)60576-6

[3] Luo, D. *et al.* Thermal regime of warm-dry permafrost in relation to ground surface temperature in the Source Areas of the Yangtze and Yellow rivers on the Qinghai-Tibet Plateau, SW China. *Sci. Total Environ.* **618**, 1033–1045 (2018). 29092743 10.1016/j.scitotenv.2017.09.083

[4] Moltumyr, L. *et al.* Long-term welfare effects of repeated warm water treatments on Atlantic salmon (*Salmo salar*). *Aquaculture* **548**, 737670. 10.1016/j.aquaculture.2021.737670 (2022).

[5] Breckels, R. D. & Neff, B. D. The effects of elevated temperature on the sexual traits, immunology and survivorship of a tropical ectotherm. *J. Exp. Biol.* **216**, 2658–2664. 10.1242/jeb.084962 (2013). 23531818 10.1242/jeb.084962

[6] Lau, E. T. C., Yung, M. M. N., Karraker, N. E. & Leung, K. M. Y. Is an assessment factor of 10 appropriate to account for the variation in chemical toxicity to freshwater ectotherms under different thermal conditions?. *Environ. Sci. Pollut. Res.* **21**, 95–104. 10.1007/s11356-013-1708-8 (2014). 10.1007/s11356-013-1708-8 23640388

[7] Li, P., Li, Z.-H. & Wu, Y. Interactive effects of temperature and mercury exposure on the stress-related responses in the freshwater fish Ctenopharyngodon idella. *Aquacult. Res.* **52**, 2070–2077. 10.1111/are.15058 (2021).

[8] Vicentini, M. *et al.* Different response of females and males Neotropical catfish (*Rhamdia quelen*) upon short-term temperature increase. *Fish Physiol. Biochem.*10.1007/s10695-023-01278-2 (2023). 38112904 10.1007/s10695-023-01278-2

[9] Klein, R. D. *et al.* Effects of increasing temperature on antioxidant defense system and oxidative stress parameters in the Antarctic fish Notothenia coriiceps and Notothenia rossii. *J. Thermal Biol.* **68**, 110–118. 10.1016/j.jtherbio.2017.02.016 (2017). 10.1016/j.jtherbio.2017.02.016 28689712

[10] Schleger, I. C. *et al.* Cold and warm waters: Energy metabolism and antioxidant defenses of the freshwater fish Astyanax lacustris (Characiformes: Characidae) under thermal stress. *J. Comparative Physiol. B* **192**, 77–94. 10.1007/s00360-021-01409-2 (2022). 10.1007/s00360-021-01409-2 34591144

[11] Schoonen, M. A. A., Xu, Y. & Strongin, D. R. An introduction to geocatalysis. *J. Geochem. Exploration* **62**, 201–215. 10.1016/s0375-6742(97)00069-1 (1998).

[12] Moustafa, M. I. Mineralogical characteristics of the separated magnetic rutile of the Egyptian Black Sands. *Resource Geol.* **60**, 300–312. 10.1111/j.1751-3928.2010.00130.x (2010).

[13] Moustafa, M. Mineralogy and beneficiation of some economic minerals in the Egyptian black sands. *Ph. D. Thesis, Fac. Sci. Mansoura University* (1999).

[14] Awad, M. *et al.* Radioactive risk assessment of beach sand along the coastline of Mediterranean Sea at El-Arish area, North Sinai, Egypt. *Mar. Pollut. Bull.* **177**, 113494. 10.1016/j.marpolbul.2022.113494 (2022). 35245768 10.1016/j.marpolbul.2022.113494

[15] Aziz, A., Sief, R., Ghieth, B. & Kaiser, M. Black sand deposits; their spatial distribution and hazards along the northern coast of Sinai Peninsula, Egypt. *J. Appl. Geophys.* **183**, 104219. 10.1016/j.jappgeo.2020.104219 (2020).

[16] Hilal, M. A. & Borai, E. H. Hazardous parameters associated with natural radioactivity exposure from black sand. *Regulat. Toxicol. Pharmacol.* **92**, 245–250. 10.1016/j.yrtph.2017.12.014 (2018). 10.1016/j.yrtph.2017.12.014 29277438

[17] Ibrahim, T., Abu Halawa, A., Ali, K. & Gaafar, I. Occurrence of black sand deposits on the Red Sea coastal plain of Wadi Diit, South Eastern Desert, Egypt: A preliminary study. *Sed. Egypt* **17**, 107–116 (2009).

[18] Ibrahim, T.* et al.* in *Petrogenesis and Exploration of the Earth’s Interior.* (eds D.M. Doronzo, E. Schingaro, J.S. Armstrong-Altrin, & B. Zoheir) 129–132 (Springer International Publishing).

[19] Kaiser, M. F., Aziz, A. M. & Ghieth, B. M. Environmental hazards and distribution of radioactive black sand along the Rosetta coastal zone in Egypt using airborne spectrometric and remote sensing data. *J. Environ. Radioact.* **137**, 71–78. 10.1016/j.jenvrad.2014.06.006 (2014). 25011074 10.1016/j.jenvrad.2014.06.006

[20] Saad, E. & Sayed, A. E.-D. H. Effects of black sand on Oreochromis niloticus: Insights into the biogeochemical impacts through an experimental study. *Front. Earth Sci.*10.3389/feart.2023.1289665 (2023).

[21] Abdelbaki, R. F., Hakamy, A., Afify, N., Abd El-Aal, M. & Abd-Elnaiem, A. M. Structural, optical, photocatalytic, and magnetic properties of new hydrothermal synthesized Cd_1__−__x_Sn_x_Fe_2_O_4_ nanocomposites. *Inorgan. Chem. Commun.* **160**, 111861. 10.1016/j.inoche.2023.111861 (2024).

[22] Sellami, B. *et al.* Impacts of nanoparticles and phosphonates in the behavior and oxidative status of the mediterranean mussels (Mytilus galloprovincialis). *Saudi J. Biol. Sci.* **28**, 6365–6374. 10.1016/j.sjbs.2021.07.017 (2021). 34764754 10.1016/j.sjbs.2021.07.017 PMC8568998

[23] Oberdörster, G., Stone, V. & Donaldson, K. Toxicology of nanoparticles: A historical perspective. *Nanotoxicology* **1**, 2–25. 10.1080/17435390701314761 (2007).

[24] Wu, J. *et al.* Toxicity and penetration of TiO2 nanoparticles in hairless mice and porcine skin after subchronic dermal exposure. *Toxicol. Lett.* **191**, 1–8. 10.1016/j.toxlet.2009.05.020 (2009). 19501137 10.1016/j.toxlet.2009.05.020

[25] Ghosh, M., Bandyopadhyay, M. & Mukherjee, A. Genotoxicity of titanium dioxide (TiO2) nanoparticles at two trophic levels: Plant and human lymphocytes. *Chemosphere* **81**, 1253–1262. 10.1016/j.chemosphere.2010.09.022 (2010). 20884039 10.1016/j.chemosphere.2010.09.022

[26] Abdel Rahman, A. N. *et al.* Silica nanoparticles acute toxicity alters ethology, neuro-stress indices, and physiological status of African catfish (Clarias gariepinus). *Aquacult. Rep.* **23**, 101034. 10.1016/j.aqrep.2022.101034 (2022).

[27] Rashidian, G. *et al.* Long-term exposure to small-sized silica nanoparticles (SiO2-NPs) induces oxidative stress and impairs reproductive performance in adult zebrafish (Danio rerio). *Comparative Biochem. Physiol. Part C Toxicol. Pharmacol.* **273**, 109715. 10.1016/j.cbpc.2023.109715 (2023). 10.1016/j.cbpc.2023.109715 37595938

[28] Lyu, K., Cao, C., Li, D., Akbar, S. & Yang, Z. The thermal regime modifies the response of aquatic keystone species Daphnia to microplastics: Evidence from population fitness, accumulation, histopathological analysis and candidate gene expression. *Sci. Total Environ.* **783**, 147154. 10.1016/j.scitotenv.2021.147154 (2021). 34088136 10.1016/j.scitotenv.2021.147154

[29] Andrade, M. *et al.* The impacts of warming on the toxicity of carbon nanotubes in mussels. *Mar. Environ. Res.* **145**, 11–21. 10.1016/j.marenvres.2019.01.013 (2019). 30771907 10.1016/j.marenvres.2019.01.013

[30] Baag, S., Ahammed, N., De, S. & Mandal, S. Combined impact of elevated temperature and zinc oxide nanoparticles on physiological stress and recovery responses of Scylla serrata. *Comparative Biochem. Physiol. Part C Toxicol. Pharmacol.* **275**, 109764. 10.1016/j.cbpc.2023.109764 (2024). 10.1016/j.cbpc.2023.109764 37827393

[31] Hamed, M. *et al.* Exposure to pyrogallol impacts the hemato-biochemical endpoints in catfish (Clarias gariepinus). *Environ. Pollut.* **333**, 122074. 10.1016/j.envpol.2023.122074 (2023). 37331582 10.1016/j.envpol.2023.122074

[32] Mohamed, I. A. *et al.* Multi-biomarkers approach to assess the toxicity of novel insecticide (Voliam flexi®) on Clarias gariepinus: From behavior to immunotoxicity. *Fish Shellfish Immunol.* **125**, 54–64. 10.1016/j.fsi.2022.04.051 (2022). 35525411 10.1016/j.fsi.2022.04.051

[33] OECD. *Test No. 203: Fish, Acute Toxicity Test*. (2019).

[34] Khieokhajonkhet, A. *et al.* Effects of long-term exposure to high temperature on growth performance, chemical composition, hematological and histological changes, and physiological responses in hybrid catfish [♂ Clarias gariepinus (Burchell, 1822) × ♀ C. macrocephalus (Günther, 1864)]. *J. Thermal Biol.* **105**, 103226 (2022). 10.1016/j.jtherbio.2022.103226 35393060

[35] Wilson, M. M., Peng, J., Dabiri, J. O. & Eldredge, J. D. Lagrangian coherent structures in low Reynolds number swimming. *J. Phys. Condensed Matter* **21**, 204105 (2009). 21825514 10.1088/0953-8984/21/20/204105

[36] Hamed, M., Said, R. E. M., Soliman, H. A. M., Osman, A. G. M. & Martyniuk, C. J. Immunotoxicological, histopathological, and ultrastructural effects of waterborne pyrogallol exposure on African catfish (Clarias gariepinus). *Chemosphere* **349**, 140792. 10.1016/j.chemosphere.2023.140792 (2024). 38016523 10.1016/j.chemosphere.2023.140792

[37] Sayed, A.E.-D.H., Hamed, M., El-Sayed, A. A. A., Nunes, B. & Soliman, H. A. M. The mitigating effect of Spirulina (Arthrospira platensis) on the hemotoxicity of gibberellic acid on juvenile tilapia (Oreochromis niloticus). *Environ. Sci. Pollut. Res.* **30**, 25701–25711. 10.1007/s11356-022-23844-6 (2023). 10.1007/s11356-022-23844-6 PMC9995583 36346524

[38] Hamed, M., Soliman, H. A. & Sayed, A.E.-D.H. Ameliorative effect of Spirulina platensis against lead nitrate–induced cytotoxicity and genotoxicity in catfish Clarias gariepinus. *Environ. Sci. Pollut. Res.* **26**, 20610–20618 (2019). 10.1007/s11356-019-05319-3 31104244

[39] Aebi, H. *Methods in Enzymology* Vol. 105, 121–126 (Academic Press, 1984). 10.1016/s0076-6879(84)05016-3 6727660

[40] Nishikimi, M., Appaji Rao, N. & Yagi, K. The occurrence of superoxide anion in the reaction of reduced phenazine methosulfate and molecular oxygen. *Biochem. Biophys. Res. Commun.* **46**, 849–854. 10.1016/S0006-291X(72)80218-3 (1972). 4400444 10.1016/s0006-291x(72)80218-3

[41] Koracevic, D., Koracevic, G., Djordjevic, V., Andrejevic, S. & Cosic, V. Method for the measurement of antioxidant activity in human fluids. *J. Clin. Pathol.* **54**, 356–361. 10.1136/jcp.54.5.356 (2001). 11328833 10.1136/jcp.54.5.356 PMC1731414

[42] Knedel, M. & Böttger, R. A kinetic method for determination of the activity of pseudocholinesterase (acylcholine acyl-hydrolase 3.1. 1.8.). *Klinische Wochenschrift* **45**, 325–327 (1967). 5588017 10.1007/BF01747115

[43] Foster, L. B. & Dunn, R. T. Single-antibody technique for radioimmunoassay of cortisol in unextracted serum or plasma. *Clin. Chem.* **20**, 365–368 (1974). 4813393

[44] Fischer, A., Jacobson, K., Rose, J. & Zeller, R. Hematoxylin and eosin staining of tissue and cell sections. *CSH Protocols.* **2008**, pdb.prot4986. 10.1101/pdb.prot4986 (2008). 21356829 10.1101/pdb.prot4986

[45] McManus, J. Histological demonstration of mucin after periodic acid. *Nature* **158**, 202–202 (1946). 20995486 10.1038/158202a0

[46] Huang, Y. *et al.* Image analysis of liver collagen using sirius red is more accurate and correlates better with serum fibrosis markers than trichrome. *Liver Int.* **33**, 1249–1256. 10.1111/liv.12184 (2013). 23617278 10.1111/liv.12184

[47] Li, X.-S., Zhu, G.-T., Luo, Y.-B., Yuan, B.-F. & Feng, Y.-Q. Synthesis and applications of functionalized magnetic materials in sample preparation. *TrAC Trends Anal. Chem.* **45**, 233–247. 10.1016/j.trac.2012.10.015 (2013).

[48] Prasdiantika, R., Susanto, S. & Kusumawardani, Y. Synthesis and Characterization of Triamine modified coated Iron Sand Hybrid Nanomaterials originating from Kendal Coast. *Jurnal Kimia Sains dan Aplikasi* **23**, 68–74. 10.14710/jksa.23.3.68-74 (2020).

[49] Hamed, M. *et al.* Occurrence, distribution, and composition of black sand along the Red Sea, Egypt. *Sci. Total Environ.*10.1016/j.scitotenv.2024.171277 (2024). 38408651 10.1016/j.scitotenv.2024.171277

[50] Aldosari, B. N. *et al.* Synthesis and characterization of magnetic Ag–Fe3O4@polymer hybrid nanocomposite systems with promising antibacterial application. *Drug Develop. Ind. Pharmacy* **49**, 723–733. 10.1080/03639045.2023.2277812 (2023). 10.1080/03639045.2023.2277812 37906615

[51] Ali, H., Ibrahim, S., Abo Zeid, E., Al-Hossainy, A. & Abd El-Aal, M. A comparative study of Cu-anchored 0D and 1D ZnO nanostructures for the reduction of organic pollutants in water. *RSC Adv.* **12**, 16496–16509. 10.1039/D2RA02515A (2022). 35754865 10.1039/d2ra02515a PMC9168830

[52] Mousa, H. *et al.* Development of environmentally friendly catalyst Ag-ZnO@cellulose acetate derived from discarded cigarette butts for reduction of organic dyes and its antibacterial applications. *Int. J. Biol. Macromol.* **258**, 128890. 10.1016/j.ijbiomac.2023.128890 (2024). 38134996 10.1016/j.ijbiomac.2023.128890

[53] Sani Gano, Z., Akuaden Audu, E., Ayoola Osigbesan, A., Femi Ade-Ajayi, A. & Barminas, J. T. Novel mesoporous iron oxide synthesized from naturally occurring magnetic sand: A potential and promising catalyst for chemical processes. *Inorg. Chem. Commun.* **159**, 111854. 10.1016/j.inoche.2023.111854 (2024).

[54] Bakr, Z. *et al.* Toxicity of silver, copper oxide, and polyethylene nanoparticles on the earthworm *Allolobophora caliginosa* using multiple biomarkers. *Appl. Soil Ecol.* **181**, 104681. 10.1016/j.apsoil.2022.104681 (2023).

[55] ATSDR, U. (1999).

[56] Doyle, J., Harper, C., Keith, S., Mumtaz, M. & Tarragó, O. Toxicological profile for radon. (2012). 24049860

[57] Shuaibu, H. K., Khandaker, M. U., Alrefae, T. & Bradley, D. A. Assessment of natural radioactivity and gamma-ray dose in monazite rich black Sand Beach of Penang Island, Malaysia. *Mar. Pollut. Bull.* **119**, 423–428. 10.1016/j.marpolbul.2017.03.026 (2017). 28342594 10.1016/j.marpolbul.2017.03.026

[58] Atteya, T. E. M. & Abbas, F. Testing a stepped solar still with different sand beds and reflectors. *Case Stud. Thermal Eng.* **43**, 102782. 10.1016/j.csite.2023.102782 (2023).

[59] Omara, Z. M. & Kabeel, A. E. The performance of different sand beds solar stills. *Int. J. Green Energy* **11**, 240–254. 10.1080/15435075.2013.769881 (2014).

[60] Kingsolver, J. G. & Woods, H. A. Beyond thermal performance curves: modeling time-dependent effects of thermal stress on ectotherm growth rates. *Am. Naturalist* **187**, 283–294. 10.1086/684786 (2016). 26913942 10.1086/684786

[61] Overton, K. *et al.* Salmon lice treatments and salmon mortality in Norwegian aquaculture: A review. *Rev. Aquacult.* **11**, 1398–1417. 10.1111/raq.12299 (2019).

[62] Elsheshtawy, A. *et al.* Exploring the impact of thermal delousing on gill health and microbiome dynamics in farmed Atlantic Salmon. *Aquaculture* **582**, 740455. 10.1016/j.aquaculture.2023.740455 (2024).

[63] Abdel-Khalek, A. A., Badran, S. R. & Marie, M.-A.S. The effective adsorbent capacity of rice husk to iron and aluminum oxides nanoparticles using Oreochromis niloticus as a bioindicator: biochemical and oxidative stress biomarkers. *Environ. Sci. Pollut. Res.* **27**, 23159–23171. 10.1007/s11356-020-08906-x (2020). 10.1007/s11356-020-08906-x 32333341

[64] Dawood, M. A. O., Noreldin, A. E. & Sewilam, H. Blood biochemical variables, antioxidative status, and histological features of intestinal, gill, and liver tissues of African catfish (Clarias gariepinus) exposed to high salinity and high-temperature stress. *Environ. Sci. Pollut. Res.* **29**, 56357–56369. 10.1007/s11356-022-19702-0 (2022). 10.1007/s11356-022-19702-0 PMC9374635 35338459

[65] Abdel-Khalek, A. A., Al-Quraishy, S. & Abdel-Gaber, R. Evaluation of nephrotoxicity in oreochromis niloticus after exposure to aluminum oxide nanoparticles: Exposure and recovery study. *Bull. Environ. Contamination Toxicol.* **108**, 292–299. 10.1007/s00128-021-03335-z (2022). 10.1007/s00128-021-03335-z 34331072

[66] Ali, A. *et al.* Exploring the impact of silica and silica-based nanoparticles on serological parameters, histopathology, organ toxicity, and genotoxicity in Rattus norvegicus. *Appl. Surface Sci. Adv.* **19**, 100551. 10.1016/j.apsadv.2023.100551 (2024).

[67] Luft, F. C. Biomarkers and predicting acute kidney injury. *Acta Physiol.* **231**, e13479. 10.1111/apha.13479 (2021). 10.1111/apha.13479 32311830

[68] Kanu, K. C., Okoboshi, A. C. & Otitoloju, A. A. Haematological and biochemical toxicity in freshwater fish Clarias gariepinus and Oreochromis niloticus following pulse exposure to atrazine, mancozeb, chlorpyrifos, lambda-cyhalothrin, and their combination. *Comparative Biochem. Physiol. Part C Toxicol. Pharmacol.* **270**, 109643. 10.1016/j.cbpc.2023.109643 (2023). 10.1016/j.cbpc.2023.109643 37137385

[69] Mahmoud, U. M., Mekkawy, I. A. A., Naguib, M. & Sayed, A.E.-D.H. Silver nanoparticle–induced nephrotoxicity in Clarias gariepinus: Physio-histological biomarkers. *Fish Physiol. Biochem.* **45**, 1895–1905. 10.1007/s10695-019-00686-7 (2019). 31399920 10.1007/s10695-019-00686-7

[70] Yin, P. *et al.* Environmentally driven changes in Atlantic salmon oxidative status interact with physiological performance. *Aquaculture* **581**, 740400. 10.1016/j.aquaculture.2023.740400 (2024).

[71] Schulte, P. M. The effects of temperature on aerobic metabolism: Towards a mechanistic understanding of the responses of ectotherms to a changing environment. *J. Exp. Biol.* **218**, 1856–1866. 10.1242/jeb.118851 (2015). 26085663 10.1242/jeb.118851

[72] Li, S. *et al.* Physiological responses to heat stress in the liver of rainbow trout (Oncorhynchus mykiss) revealed by UPLC-QTOF-MS metabolomics and biochemical assays. *Ecotoxicol. Environ. Safety* **242**, 113949. 10.1016/j.ecoenv.2022.113949 (2022). 35999764 10.1016/j.ecoenv.2022.113949

[73] Madeira, D., Narciso, L., Cabral, H. N., Vinagre, C. & Diniz, M. S. Influence of temperature in thermal and oxidative stress responses in estuarine fish. *Comparative Biochem. Physiol. Part A Mol. Integr. Physiol.* **166**, 237–243. 10.1016/j.cbpa.2013.06.008 (2013). 10.1016/j.cbpa.2013.06.008 23774589

[74] Waheed, R. *et al.* Thermal stress accelerates mercury chloride toxicity in Oreochromis niloticus via up-regulation of mercury bioaccumulation and HSP70 mRNA expression. *Sci. Total Environ.* **718**, 137326. 10.1016/j.scitotenv.2020.137326 (2020). 32092518 10.1016/j.scitotenv.2020.137326

[75] Schreck, C. B., Tort, L., Farrell, A. & Brauner, C. *Biology of Stress in Fish* (Academic Press, 2016).

[76] Almeida, J. R., Oliveira, C., Gravato, C. & Guilhermino, L. Linking behavioural alterations with biomarkers responses in the European seabass Dicentrarchus labrax L. exposed to the organophosphate pesticide fenitrothion. *Ecotoxicology (London, England)* **19**, 1369–1381. 10.1007/s10646-010-0523-y (2010). 20686920 10.1007/s10646-010-0523-y

[77] Hamed, M. *et al.* Oxidative stress, antioxidant defense responses, and histopathology: Biomarkers for monitoring exposure to pyrogallol in Clarias gariepinus. *J. Environ. Manag.* **351**, 119845 (2024). 10.1016/j.jenvman.2023.119845 38109825

[78] Sayed, A.E.-D.H. *et al.* Climate change induce the toxicity of black sand nanoparticles on catfish (*Clarias gariepinus*) using hemato-hepatological biomarkers. *BioNanoScience.*10.1007/s12668-024-01549-z (2024).

[79] Chatterjee, N. & Bhattacharjee, B. Changing physicochemical properties of water due to exposure of ZnS nanoparticles and its detrimental effect on feeding behaviour and liver of a non-air breathing catfish Mystus vittatus. *Int. J. Latest Res. Sci. Technol.* **3**, 199–204 (2014).

[80] Naguib, M., Mahmoud, U. M., Mekkawy, I. A. & Sayed, A.E.-D.H. Hepatotoxic effects of silver nanoparticles on Clarias gariepinus; Biochemical, histopathological, and histochemical studies. *Toxicol. Rep.* **7**, 133–141. 10.1016/j.toxrep.2020.01.002 (2020). 31956514 10.1016/j.toxrep.2020.01.002 PMC6962648

[81] Almanaa, T. N. *et al.* Silica nanoparticle acute toxicity on male *Rattus norvegicus* domestica: Ethological behavior, hematological disorders, biochemical analyses, hepato-renal function, and antioxidant-immune response. *Front. Bioeng. Biotechnol.*10.3389/fbioe.2022.868111 (2022). 35464726 10.3389/fbioe.2022.868111 PMC9022119

[82] Zaman, M. *et al.* Physiological and histopathological effects of polystyrene nanoparticles on the filter-feeding fish Hypophthalmichthys molitrix. *Sci. Total Environ.* **912**, 169376. 10.1016/j.scitotenv.2023.169376 (2024). 38104827 10.1016/j.scitotenv.2023.169376

[83] Monfared, A. L., Bahrami, A. M., Hosseini, E., Soltani, S. & Shaddel, M. Retracted article: Effects of nano-particles on histo-pathological changes of the fish. *J. Environ. Health Sci. Eng.* **13**, 62. 10.1186/s40201-015-0216-9 (2015). 26322234 10.1186/s40201-015-0216-9 PMC4552984

[84] Hoseini, S. M., Hedayati, A., Taheri Mirghaed, A. & Ghelichpour, M. Toxic effects of copper sulfate and copper nanoparticles on minerals, enzymes, thyroid hormones and protein fractions of plasma and histopathology in common carp Cyprinus carpio. *Exp. Toxicol. Pathol.* **68**, 493–503. 10.1016/j.etp.2016.08.002 (2016). 27555376 10.1016/j.etp.2016.08.002

[85] Haghighat, F., Kim, Y., Sourinejad, I., Yu, I. J. & Johari, S. A. Titanium dioxide nanoparticles affect the toxicity of silver nanoparticles in common carp (Cyprinus carpio). *Chemosphere* **262**, 127805. 10.1016/j.chemosphere.2020.127805 (2021). 32750593 10.1016/j.chemosphere.2020.127805

[86] Ates, M. *et al.* Chronic exposure of tilapia (Oreochromis niloticus) to iron oxide nanoparticles: Effects of particle morphology on accumulation, elimination, hematology and immune responses. *Aquatic Toxicol.* **177**, 22–32. 10.1016/j.aquatox.2016.05.005 (2016). 10.1016/j.aquatox.2016.05.005 PMC4967404 27232508

[87] Jovanović, B., Whitley, E. M., Kimura, K., Crumpton, A. & Palić, D. Titanium dioxide nanoparticles enhance mortality of fish exposed to bacterial pathogens. *Environ. Pollution* **203**, 153–164. 10.1016/j.envpol.2015.04.003 (2015). 10.1016/j.envpol.2015.04.003 25884347

[88] Li, A. J. *et al.* Extreme cold or warm events can potentially exacerbate chemical toxicity to the marine medaka fish Oryzias melastigma. *Aquatic Toxicol.* **249**, 106226. 10.1016/j.aquatox.2022.106226 (2022). 10.1016/j.aquatox.2022.106226 35738209

[89] Blewett, T. A., Wood, C. M. & Glover, C. N. Salinity-dependent nickel accumulation and effects on respiration, ion regulation and oxidative stress in the galaxiid fish, Galaxias maculatus. *Environ. Pollut.* **214**, 132–141. 10.1016/j.envpol.2016.04.010 (2016). 27077552 10.1016/j.envpol.2016.04.010

[90] El Euony, O. I., Elblehi, S. S., Abdel-Latif, H. M., Abdel-Daim, M. M. & El-Sayed, Y. S. Modulatory role of dietary Thymus vulgaris essential oil and Bacillus subtilis against thiamethoxam-induced hepatorenal damage, oxidative stress, and immunotoxicity in African catfish (Clarias garipenus). *Environ. Sci. Pollution Res.* **27**, 23108–23128. 10.1007/s11356-020-08588-5 (2020). 10.1007/s11356-020-08588-5 32333347

[91] Beegam, A. *et al.* Multiorgan histopathological changes in the juvenile seabream Sparus aurata as a biomarker for zinc oxide particles toxicity. *Environ. Sci. Pollution Res.* **27**, 30907–30917. 10.1007/s11356-019-05949-7 (2020). 10.1007/s11356-019-05949-7 31376128

[92] Zhang, T. *et al.* Non-target toxic effects of avermectin on carp spleen involve oxidative stress, inflammation, and apoptosis. *Pesticide Biochem. Physiol.* **187**, 105190. 10.1016/j.pestbp.2022.105190 (2022). 10.1016/j.pestbp.2022.105190 36127050

[93] Komoike, Y., Nomura-Komoike, K. & Matsuoka, M. Intake of acrylamide at the dietary relevant concentration causes splenic toxicity in adult zebrafish. *Environ. Res.* **189**, 109977. 10.1016/j.envres.2020.109977 (2020). 32980030 10.1016/j.envres.2020.109977

[94] Lyu, L. *et al.* Novel insights into DEHP-induced zebrafish spleen damage: Cellular apoptosis, mitochondrial dysfunction, and innate immunity. *Sci. Total Environ.* **912**, 169324. 10.1016/j.scitotenv.2023.169324 (2024). 38145680 10.1016/j.scitotenv.2023.169324

[95] Montes, M. O., Hanna, S. K., Lenihan, H. S. & Keller, A. A. Uptake, accumulation, and biotransformation of metal oxide nanoparticles by a marine suspension-feeder. *J. Hazardous Mater.* **225–226**, 139–145. 10.1016/j.jhazmat.2012.05.009 (2012). 10.1016/j.jhazmat.2012.05.009 22614026

